# ACTIVITY OF *ORBIGNYA PHALERATA* AND *EUTERPE EDULES* IN THE PREVENTION AND TREATMENT OF PEPTIC ULCER IN RATS

**DOI:** 10.1590/0102-672020180001e1390

**Published:** 2018-08-16

**Authors:** Orlando Jorge Martins TORRES, Orlando José dos SANTOS, Roberto Soares de MOURA, Humberto Oliveira SERRA, Vanisse Portela RAMOS, Syomara Pereira da Costa MELO, Carlos Manoel Bulcão LOUREIRO

**Affiliations:** 1 Department of Surgery, Federal University of Maranhão, São Luís, MA;; 2 Department of Pharmacology, State University of Rio de Janeiro, Rio de Janeiro, RJ;; 3 Department of Pathology, Federal University of Maranhão, São Luís, MA;; 4 Department of Surgery, Hospital São Domingos of Maranhão, São Luís, MA, Brazil

**Keywords:** Peptic ulcer, Plants, medicinal, Rats, Úlcera péptica. Plantas, medicinais. Ratos

## Abstract

**Background:**

Peptic ulcer is considered a public health problem associated with loss of quality of life. Does not exist optimal therapeutic regimen. The search for alternative treatments using foods or plants that may assist in gastric protection may become marked in this population because of their easy access and low cost.

**Aim:**

To study the antiulcerogenic activity of extracts of Orbignya phalerata (babaçu) and Euterpe edules (juçara) in Wistar rats after induction of peptic ulcer, compared with Omeprazole.

**Method:**

Forty Wistar rats were distributed into four groups: group I, II, III, IV (10 rats each) subjected to extract of Orbignya phalerata, Euterpe edules, Omeprazole and ethanol, respectively. Each group of 10 rats was divided into subgroups of five for prophylaxis and therapeutic study.

**Results:**

The pre-treatment with juçara extract has provided a significant protection against peptic ulcer induced by ethanol. In the prophylactic subgroup, Omeprazole resulted in protection. In addition to protection against peptic ulcer, inflammation and neocapillarization were also variables with a statistical significance in the prophylaxis subgroups using omeprazole and juçara. In the therapeutic subgroup, omeprazole, juçara and babaçu were statistically different as for protection against the presence of inflammation and the healing of ulcers.

**Conclusion:**

The extracts of juçara and babaçu behaved as the omeprazole, evidencing the therapeutic activity of these extracts.

## RESUMO

**Racional:**

A úlcera péptica é considerada problema de saúde pública, associada a perda na qualidade de vida. Não existe esquema terapêutico ideal. A busca de tratamentos alternativos, com uso de alimentos ou plantas que possam ajudar na proteção gástrica, torna-se viável à população, por ser de fácil acesso e baixo custo.

**Objetivo:**

Estudar a atividade antiulcerogênica dos extratos de Orbignya phalerata (babaçu) e Euterpe edules (juçara) em ratos Wistar após indução de úlcera péptica e comparar com omeprazol.

**Método:**

Quarenta ratos Wistar foram distribuídos em quatro grupos: grupo I, II, III, IV (10 ratos) submetidos ao extrato de Orbignya phalerata, Euterpe edules, omeprazol e álcool etílico, respectivamente. Cada grupo de 10 ratos foi dividido em subgrupos de cinco para estudar a profilaxia e tratamento.

**Resultados:**

O pré-tratamento com extrato de juçara forneceu proteção significativa contra a ulceração péptica induzida pelo etanol, assim como o omeprazol. Além da proteção contra úlcera péptica, inflamação e neocaplilarização também foram variáveis com significância estatística nos subgrupos profilaxia do omeprazol e da juçara. No subgrupo terapêutico, o omeprazol, juçara e babaçu teve diferença estatística para proteção contra a presença de inflamação e cicatrização de úlcera.

**Conclusão:**

Os extratos de juçara e babaçu se comportaram como omeprazol, evidenciando o poder terapêutico desses extratos.

## INTRODUCTION

Ulcer is the term used to denote open lesions with tissue loss. Peptic ulcers are defects in the gastrointestinal mucosa extending through the mucosa and muscular - the thin layer of smooth muscle found in much of the digestive tract (stomach and/or duodenum) - located between the lamina proper and the submucosa^1^. Peptic ulcer has been identified as the main disease of the 21^st^ century due to profound changes in life habits, mainly feeding habits^1^.

It is estimated that, in Brazil, the prevalence of ulcers in men is 0.2% and in women 0.1%. The national mortality rate is estimated at 3.0/100 thousand inhabitants (3.6/100 thousand for men, 2.3/100 thousand for women)^13^. The treatment aims to relieve pain, manage ulcer healing and prevent complications and recurrence. Although there is no optimal therapeutic pharmacological scheme, the effective scheme consists of a proton pump inhibitor^3^.

However, the number of relapses is still high, and there are side effects. Therefore, the use of medicinal plants is increasingly widespread as an alternative treatment to current therapy. The Amazon has 50% of the planet’s biodiversity, according to data of research institutions in the region. About 5,000 of the 25,000 Amazonian species have already been cataloged and their therapeutic properties studied. From a scientific point of view, however, it is still a field that has not been studied and widespread in the country^7^ ^,^ ^9^ ^,^ ^14^.

This paper researched the extracts of babaçu and juçara. Babaçu, also known locally as “bauçu, baguaçu, auaçu, aguaçu, guaguaçu, uauaçu, coco-de-monaco, coco-de-palmeira, coco-naia, coco-pindoba and palha-branca”, is a palm native to the north of Brazil. Its highest concentration is in the State of Maranhão. Piauí, Pará and Tocantins states are also major domestic producers^1^ ^,^ ^2^ ^,^ ^5^ ^,^ ^6^ ^,^ ^16^. Its leaf straws are transformed into baskets, the bark of the coconut is transformed into charcoal and the chestnut in oil and soap. The powder of the babaçu coconut mesocarp is popularly known as starch, and has been used as food and as a medicament because it has anti-inflammatory, immunomodulatory, analgesic and antipyretic activities^1^ ^,^ ^2^ ^,^ ^16^.

Juçara is a palm tree native to the Atlantic Forest, found from Rio Grande do Sul to the south of Bahia. It is also known as “Jiçara, Içara or Ripeira” due to the traditional use of its stem for the production of stacks and beams for construction. The “Palmiteiro” or “Palmito Juçara” is widely cultivated due to its use for the production of canned palm heart, widely distributed, appreciated and consumed in large urban centers^14^. With the increasing demand for palm heart consumption since the 1950s and 1960s, this species has been heavily exploited in native forests. The production of palm heart implies cutting the plant, causing its death. Because of this, juçara is today a threatened species^14^.

In this study, the mesocarp of the babaçu (*Orbignya phalerata*) and the juçara pulp (*Euterpe edulis*) were studied aiming to evaluate the protective and therapeutic effects after induction of peptic ulcer in experimental models.

The use of plants to prevent and treat diseases is a millenary practice^6^ ^,^ ^13^ ^,^ ^16^. A number of studies were conducted using plants, demonstrating that they may be useful for the treatment of peptic ulcers of humans and animal models by means of different mechanisms^6^ ^,^ ^11^ ^,^ ^13^. 

The aim of this study was to study the antiulcer activity of extracts of *Orbignya phalerata* and *Euterpe edules* and to analyze prophylactic and therapeutic effects with omeprazole after induction of peptic ulcer in rats.

## METHODS

### Animals and experimental environment

The sample was composed of 16 male Wistar rats (*Ratus novergicus*), weight between 180-240 g, supplied by the Central Vivarium of the Federal University of Maranhão. At six weeks of age, the animals were transferred to the Laboratory of Experimental Surgery of Maranhão for adaptation for two weeks. They were kept in plastic boxes with a galvanized mesh lid and xylan-covered bottom. There were five rats per cage. The cages were sanitized three times per week. All animals were kept in the vivarium for 12 h daily periods under a continuous flow of air and at room temperature. All items pertaining to the experiment, such as purchase, transport, conditions of the vivarium, nutrition, veterinary care and records, followed the International Principles of Biotechnology and Biotechnology Research Involving Animals, and the researchers sought to treat animals by avoiding or minimizing discomforts, risks or pain as essential ethical imperatives. All data were recorded and entered into an Excel database specifically designed for this purpose.

### Food and water

The animals were fed from birth to 21 days of life with breastfeeding. After this period, they were fed with water and rat ration ad libitum. The ration was changed twice a week. The drinking water was supplied by the water supply network of São Luís and changed every two days.

### Vegetable material

Were used babaçu mesocarp (*Orbignya phalerata)* and juçara pulp (*Euterpe edules*). Babacu was purchased from local producers and juçara from Rio de Janeiro and bought in theCeasa market in São Luís, MA, Brasil. The products were authenticated by a qualified professional, and cataloged in the herbarium of the Federal University of Maranhão: babaçu 01371 (*Orbignya phalerata*) and juçara 01173 (*Euterpe edules*). From the babaçu was used the mesocarp (part between the seed and the shell) that when mature, when being separated, turns into powder, used in the research. Juçara had its pulp used (part comprised between the seed and the bark, separated after being kneaded). These products were diluted in a ratio of 1 g of mass to 3 ml of distilled water and left for extraction for 24 h. Subsequently, they were subjected to three filtrations for three consecutive days, for obtaining aqueous extracts. It was used in the first two days, cloth strainer and in the last filtrate (content obtained after the filtrations) a suction pump for filtration was used, in order to remove impurities. The last filtration was carried out using a suction pump in order to remove impurities. After preparation, these extracts were stored in dark flasks to prevent incidence of light and to reduce the oxidative action, which could alter the chemical components. The dry weight of each extract was calculated. A weighed flask was used, and 0.5ml of each extract was added, which was placed in an oven at 80^o^ C for remove all moisture.

### Experimental design

The animals were distributed into four groups of ten animals each. Group I received the extract of *Orbignya phalerata* by oral gavage at a concentration of 2.0 g/kg. Group II received the extract of *Euterpe edules* by oral gavage at a concentration of 2.0 g/kg. Group III received omeprazole by washing at a dose of 20 mg/kg (positive control). Group IV received only water, ration and ethyl alcohol at 70% (0.5 ml/200g, negative control). Each of these four groups with ten animals was subdivided into two groups of five. The aqueous extracts from medicinal plants and omeprazole were given to the first subgroups for three consecutive days. On the 4^th^ day the ulcer was induced with ethyl alcohol. On the 5^th^ day, animals were killed for gastroprotective effect testing. The other five animals of subgroups had their ulcers induced on the 1^st^ day and underwent oral administration of extracts and omeprazole for three consecutive days. On the 5^th^ day, death was induced to evaluate therapeutic effects. A total gastrectomy was performed on all animals on the 5^th^ day of experimentation. 

### Surgical procedure and sample handling

The stomachs were completely removed, opened at the great curvature and carefully washed with distilled water for withdrawal of feed and to allow a better macroscopic analysis of gastric lesions. In the macroscopic analysis, the presence of ulcer, hyperemic mucosa, loss of mucous folds and hemorrhage were evaluated. The gastric parts were fixed in Styrofoam and immersed in 10% formalin. The blades were made using hematoxylin-eosin and picrocyanus RED staining. Histopathological analysis was performed by a single professional who was unaware of the type of exposure to which the animals had been subjected. This professional, by the Service of Pathology of the University Hospital, conducted an analysis of inflammation, capillary neoformation, collagenization, re-epithelization and ulcer, and classified these variables as for presence or absence. After tabulation, the data were classified for statistical analysis.

### Statistical analysis

The data were evaluated by NCSS 11 Statical Software (2016). The non-parametric qui-square test of independence (c^2^) was used to evaluate the association of the groups with the result of the microscopic and macroscopic classifier variables.The ordinal variables (inflammation and depth) were evaluated using the Kruskall-Wallis test, followed by the Dunn test for the post hoc comparison of the medians. Comparison of the subgroups within each group was done using Fisher’s exact test. The level of significance to reject the null hypothesis was 5%, that is, a value of p<0.05 was considered statistically significant.

## RESULTS

The numerical value of the tables corresponds to the number of rats in which the mentioned characteristic was absent or present.

In the alcohol group, the 10 rats had ulcers macroscopically identified. Juçara had a lower ulcer identification than the omeprazole group, thus avoiding the prophylactic effect of this extract (p=0.015). However, for the other variables (hyperemia, loss of mucous folds and hemorrhage), the omeprazole group generated greater protection (all with statistical significance, Table 1).

**TABLE 1:** Macroscopic characteristics of gastric lesions in the prophylaxis subgroups

Group	Ulcer		Mucosal hyperemia		Loss of fold		Bleeding	
	Absent	Present	Absent	Present	Absent	Present	Absent	Present
Omeprazole	3	2	3	2	3	2	3	2
Babaçu	3	2	5	0	5	0	5	0
Juçara	5	0	5	0	5	0	5	0
Alcohol	0	5	0	5	0	5	0	5
p	0,015		0,002		0,001		0,002	

When the microscopic characteristics of necrosis and fibrosis were evaluated in the therapeutic subgroup, the juçara and babaçu groups behaved similarly to the omeprazole group with statistical significance, protecting against necrosis and fibrosis (Table 2).

**TABLE 2:** Microscopic characteristics of gastric lesions in therapeutic subgroups

Group	Necrosis		Fibrosis		Reepithelialization		Neocapilar	
	Absent	Present	Absent	Present	Absent	Present	Absent	Present
Omeprazole	4	1	5	0	5	0	5	0
Babaçu	4	1	5	0	4	1	5	0
Juçara	4	1	5	0	5	0	4	1
Alcohol	1	4	2	3	3	2	3	2
p	0,038		0,048		0,336		0,017	

The babaçu presented marked inflammation in the prophylaxis subgroup when compared to omeprazol and juçara. In relation to ulcer depth, all the animals in the alcohol control group had their ulcers reaching the submucosa, being deeper than the ulcers of the babaçu group that reached the mucosa, with statistical significance (Table 3).

**TABLE 3:** Kruskall-Wallis and Dunn’s test of ordinal variables (inflammation and depth) in relation to prophylaxis subgroups

Variable	Group	n	Medium	Dunn	p
Inflammation	Omeprazole	5	Absent	b	0,010
	Babaçu	5	Accentuated	a	
	Juçara	5	Absent	b	
	Álcool	5	Accentuated	a	
Depth	Omeprazole	5	Absent	c	0,003
	Babaçu	5	Mucosal	b	
	Juçara	5	Absent	c	
	Álcool	5	Submucosal	a	

The juçara protected against gastric ulcer formation when compared to the negative control group, alcohol. Neocapillarization was observed in both the juçara and babassu groups, with statistical significance (p=0.008, Table 4)

**TABLE 4:** Fisher’s test between groups: juçara and alcohol, babaçu and alcohol in the prophylaxis subgroup

		Group				Group			
		Juçara	Alcohol	Total	(p)	Babaçu	Alcohol	Total	p
Inflammation	No	4	0	4	0.048	1	0	1	1
	Yes	1	5	6		4	5	9	
Necrosis	No	4	2	6	0.524	5	2	7	0.167
	Yes	1	3	4		0	3	3	
Fibroblast	No	5	1	6	0.48	5	1	6	0.048
	Yes	0	4	4		0	4	4	
Fibrosis	No	5	2	7	0.167	5	2	7	0.167
	Yes	0	3	3		0	3	3	
Reepithelialization	No	5	2	7	0.167	5	2	7	0.167
	Yes	0	3	3		0	3	3	
Neocapilarizacion	No	5	0	5	0.008	5	0	5	0.008
	Yes	0	5	5		0	5	5	
Ulcer	No	4	0	4	0.048	1	0	1	1
	Yes	1	5	6		4	5	9	

## DISCUSSION

Recently, many studies have been conducted to explore new antiulcerogenic agents from natural sources. The anti-ulcer activity of several chemical compounds isolated from plants has been determined. In Brazil, several plant extracts are used in popular medicine for the treatment of digestive disorders, including gastric ulcers ^4^ ^,^ ^5^ ^,^ ^7^ ^,^ ^8^ ^,^ ^9^.

 Numerous factors such as increased vascular permeability, intestinal motility, vagal activity, decreased gastric blood flow and prostaglandin levels play an important role in the pathogenesis of gastric ulcers. Thus, several experimental models can be used in an attempt to elucidate the mechanism of action of plant extracts^4^ ^,^ ^5^ ^,^ ^8^.

 In this research, ethanol was used as a source of induction to the formation of peptic ulcer in rats. Already been described by Robert^15^, who reported that ethanol-induced ulcerations are not inhibited by substances that interfere with acid secretion, such as cimetidine, but are inhibited by agents that increase the defense factors of the mucosa, such as, for example, prostaglandins. This prompts us to define the true mechanism and the role of babaçu and juçara extracts used in this research, which present an antiulcerogenic therapeutic effect .

 In this study, all animals in the alcohol group developed ulcer after gavage, with a macroscopic perception of petechiae. As observed by Nesselo et al.^12^ in 2017, animals with gastric lesions induced by ethanol presented areas of necrosis and/or hemorrhage in the gastric mucosa, proving the toxicity of this agent. Mizui and Doteuchi^10^ defined in 1983 that the ethanol induction model evaluates, among others, the activity of cytoprotective substances. Ethanol produces necrotic lesions in the gastric mucosa.According to Nesselo et. al.^12^, one of the major mechanisms attributed to the toxic effect of ethanol is the change in gastric cell homeostasis and tissue damage resulting from a direct action on mucosa and the formation of free radicals and reactive oxygen species.As described in 2009 in a paper published by Shine et al.^17^, the authors inferred that the ethanol metabolized by the organism causes an increase in the production of O_2_ in the tissues and, simultaneously, an increase of cellular free radical concentration. These free radicals could cause DNA strand breaks and protein denaturation, resulting in gastric lesion. In this study, was performed macroscopic and microscopic analysis of the gastric parts. Macroscopic analysis with direct visualization of the stomach allows better conclusions regarding the tissue reaction in the studied groups. In this research, when the macroscopic characteristics were studied, the prophylaxis subgroups of babaçu and juçara behaved as the prophylaxis subgroup of omeprazole when the variables ulcer, hyperemic mucosa and hemorrhage were studied. 40% of animals did not develop ulcers compared to the alcohol group, in which 100% of them presented lesions. This allows us to infer that the extracts studied serve as protection against the formation of gastric lesions, such as the drug that has a recognized use for gastroprotection. When was studied macroscopic characteristics in the therapeutic subgroups, babaçu and juçara behaved as the therapeutic subgroup of omeprazol when variables ulcer and hemorrhage were studied. No animal (0%) had ulcers and hemorrhage when compared to the alcohol group, in which 100% of animals presented these lesions. It is important to emphasize that, in the therapeutic subgroup using babaçu extract, no animal that developed hyperemic mucosa had a better result than in the omeprazole group, in which 40% presented mucosal hyperemia. This allows us to infer that the babaçu extract serves as protection against the formation of mucosal hyperemia, even more than the drug already in use, i.e., omeprazole.

 Wound healing is done in three phases (inflammatory, proliferative and maturation)^16^ ^,^ ^17^. In this research, due to the study time (five days) the inflammatory and proliferative phases were the most emphasized. In the prophylactic subgroups, omeprazole and juçara prevented the formation of inflammation and neocapillarization. These variables presented a statistical significance. The juçara behaved just like the current drug of recognized efficacy, i.e., omeprazole.In the therapeutic subgroup, inflammation was studied on the 3^rd^ day after ulcer induction.

 A study published by Singer and Clark^18^ shows that the assessment of gastric healing should be made on the 3^rd^ day because it is the initial and critical phase of tissue repair. It should be expected to find an intense inflammatory process. This also happened in this research for therapeutic group.Acute inflammation to a certain degree is necessary for a good tissue repair response. However, an intense inflammatory reaction may decrease blood supply, with the consequent inhibition of fibroblast proliferation ^6,17^.

 In this study, fibroblast proliferation was not evident in either the study groups nor the subgroups. In the animals of the therapeutic subgroup, omeprazole, juçara and babaçu, a statistical difference was observed for protection against the presence of inflammation and ulcer formation. It can be observed, using the data of Tables 5 and 6, referring to the effects of omeprazole, babaçu and juçara on alcohol-induced gastric lesions in Wistar rats, that there was a statistically significant difference between the groups evaluated. 

 Thus, it is inferred that the extracts behaved as the standard drug already used for the treatment of peptic ulcers in humans, evidencing the therapeutic potential of the extracts.These results encourage future phytochemical studies on isolation and identification of the active principles of the studied extracts.

## CONCLUSION

 The extracts of juçara and babaçu behaved as the omeprazole, evidencing the therapeutic activity of these extracts. 
